# Impact of Remote Symptom Monitoring on Acute Care Utilization in Patients with Advanced Cancer Undergoing Phase I Therapies: Secondary Analysis of a Randomized Clinical Trial

**DOI:** 10.3390/cancers18213499

**Published:** 2026-10-30

**Authors:** Jack Stover, Matthew Meranda, Ishwaria M. Subbiah, Minxing Chen, Sanjay Shete, David Hong, Eduardo Bruera, David Hui

**Affiliations:** 1 Department of Palliative, Rehabilitation and Integrative Medicine, The University of Texas MD Anderson Cancer Center, Houston, TX 77030, USA; 2 Sarah Cannon Research Institute, Nashville, TN 37203, USA; 3 Department of Biostatistics, The University of Texas MD Anderson Cancer Center, Houston, TX 77030, USA; 4 Department of Investigational Cancer Therapeutics, Division of Cancer Medicine, The University of Texas MD Anderson Cancer Center, Houston, TX 77030, USA; 5 Center for Goal Concordant Care Research, The University of Texas MD Anderson Cancer Center, Houston, TX 77030, USA; 6 Department of General Oncology, The University of Texas MD Anderson Cancer Center, Houston, TX 77030, USA

**Keywords:** supportive care, early-phase clinical trials, advanced cancer, emergency department visits, hospitalizations, remote symptom monitoring

## Abstract

Purpose: Patients undergoing phase I cancer therapies experience high symptom burden and complications that may lead to emergency department (ED) visits and hospitalizations. It is unclear whether remote symptom monitoring (RSM) reduces acute care utilization. We conducted a secondary analysis of a randomized clinical trial to determine if incorporating RSM into specialist palliative care (SPC) would reduce ED visits/hospitalizations. Methods: Patients with advanced cancer undergoing phase I therapies were randomized to SPC alone or SPC+RSM. Primary outcomes were ED visit and hospitalization frequencies over 6 months. We also assessed presenting concern, presentation etiology, and potential avoidability with two independent reviewers. Results: Among 100 patients (mean age 56, 63% female, median survival 221 [95% CI 171, 271] days), 43 were in the SPC group and 57 were in the SPC+RSM group. A total of 22/43 (51%) SPC patients and 30/57 (53%) SPC+RSM patients had at least one ED visit over 6 months (*p* = 0.58), while 18/43 (42%) SPC patients and 21/57 (37%) had at least one hospitalization over 6 months (*p* = 0.56). The most common presenting symptoms were pain (ED 27%, hospitalizations 29%) and respiratory symptoms (ED 22%, hospitalization 22%). The main reasons for acute care encounters were disease progression (ED 49%, hospitalizations 58%) and acute complications (ED 26%, hospitalizations 28%). Only 4/84 (5%) ED visits and 0/57 (0%) of the hospitalizations were deemed avoidable. Conclusions: Acute care utilization was high in phase I patients. RSM did not reduce ED visits/hospitalizations, which were mostly unavoidable. Our findings highlight the need for novel strategies to support this population.

## Simple Summary

Patients with advanced cancer participating in early-phase clinical trials often experience severe symptoms and complications that lead to emergency department visits and hospitalizations. Remote symptom monitoring has been shown to improve symptom control and quality of life, but it remains unclear whether it can also reduce acute care utilization in this high-risk population. We performed a secondary analysis of a randomized controlled trial evaluating the addition of remote symptom monitoring to specialist palliative care to determine whether it reduced emergency department visits and hospitalizations and examined the reasons these events occurred. We found that acute care utilization remained high and no statistically significant difference was detected with the addition of remote symptom monitoring. Few emergency department visits and no hospitalizations were classified potentially avoidable under our retrospective assessment criteria. These findings highlight the substantial burden of acute care utilization among patients with advanced cancer participating in early-phase clinical trials and suggest that remote symptom monitoring alone may not be sufficient to reduce this risk. Additional interventions are needed to better address the medical complications that drive emergency department visits and hospitalizations in this population.

## 1. Introduction

Phase I trials evaluate safety and tolerability of novel therapeutic agents, characterize dose-limiting toxicities, and determine a recommended dose for further study. Patients with advanced cancer enrolled in phase I trials are at high risk for symptom distress and acute complications due to the advanced nature of their disease and corresponding treatment. These factors could contribute to more acute care utilization, including emergency department (ED) visits and unplanned hospitalizations. Indeed, cancer patients on phase I trials tend to have increased symptom burden compared with other cancer patients receiving treatment, even after adjusting for performance status [1,2]. Furthermore, phase I patients have often progressed on standard therapies, which exposes them to the effects of cumulative treatment toxicities in addition to the burden of advanced cancer [3,4,5]. Moreover, phase I patients tend to have a heightened interest in more intensive life-prolonging therapies, which may further drive acute care utilization [1]. Despite these many contributory factors, there has been a paucity of studies that have specifically examined the prevalence of acute care utilization in cancer patients undergoing phase I therapies [6,7].

Several strategies have been proposed to reduce acute care utilization in patients with advanced cancer, such as specialist palliative care (SPC) referral and remote symptom monitoring (RSM) [8,9,10]. SPC integration can improve symptom management and facilitate goals-of-care discussions, which can reduce the need for acute care utilization [11]. Another intervention which has shown promise in reducing ED visits and hospitalizations in cancer patients is RSM, which involves the regular, remote collection of patient symptoms, typically with corresponding thresholds of severity triggering further management by the clinical team [12,13,14,15,16]. However, given the heightened degree of monitoring phase I patients already undergo while on a clinical trial, it is unclear if additional oversight would reduce acute care utilization. 

We recently completed a randomized controlled trial evaluating the integration of RSM into palliative care for patients with advanced cancer undergoing phase I therapies versus outpatient SPC alone [17]. In that randomized controlled trial, we compared phase I cancer patients established with palliative care receiving RSM via weekly completion of an electronic symptom burden survey followed by a phone call from the palliative care team with a control group which received standard-of-care monitoring. We found that incorporating RSM resulted in a relative improvement in patient symptoms and health-related quality of life [17]. However, the effect of RSM on acute care utilization in phase I cancer patients remains poorly characterized. As such, we performed a secondary analysis evaluating the incremental benefit of RSM alongside SPC and intensive phase I monitoring in reducing acute care utilization. 

## 2. Materials and Methods

### 2.1. Study Design and Participants

**Figure 1:**
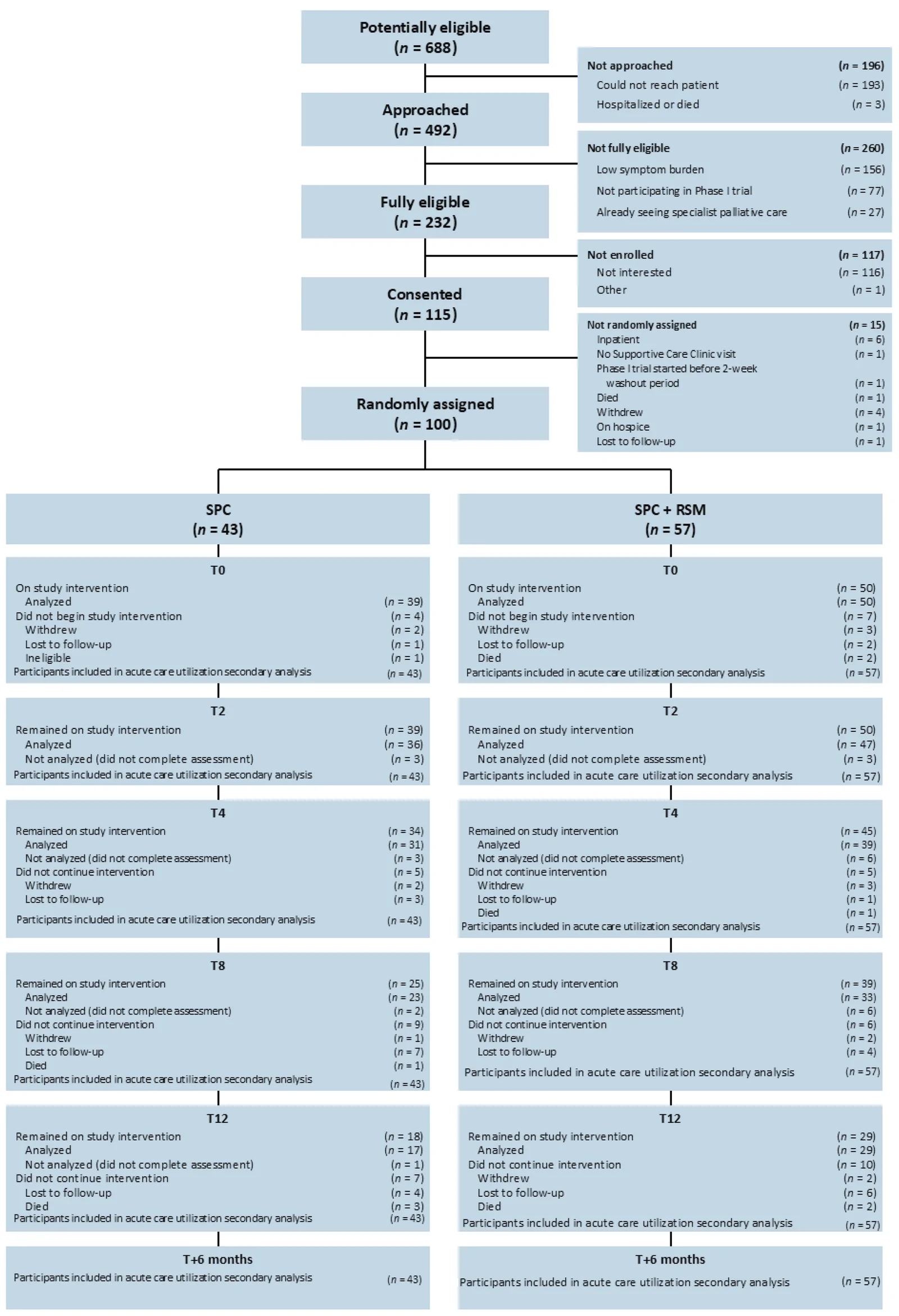
CONSORT diagram. RSM, remote monitoring; SPC, specialist palliative care.

We conducted a two parallel-group, single-blind, randomized clinical trial in patients with advanced cancer on phase I therapies examining the impact of SPC with RSM versus SPC alone on symptom control [17]. The CONSORT diagram is shown in Figure 1. Here, we performed a post hoc secondary analysis on these data to assess if RSM implementation had an impact on acute care utilization in phase I cancer patients receiving SPC+RSM versus SPC alone. The eligibility of the study participants has been previously described [17]. Briefly, patients were recruited from our targeted therapy (phase I) clinic. Inclusion criteria were age ≥ 18 at the time of phase I enrollment, diagnosis of an advanced solid tumor, moderate-to-high symptom burden (score ≥ 4/10 on at least one Edmonton Symptom Assessment System [ESAS] symptom and Global Distress Score [GDS] ≥ 20/90) and plan to start treatment with a phase I oncologic agent. Exclusion criteria included delirium (Memorial Delirium Assessment Scale [MDAS] > 8/30) and/or lack of reliable telephone/internet access [17]. This study was conducted at the University of Texas MD Anderson Cancer Center (MD Anderson, Houston, TX, USA) and approved by its Institutional Review Board. All patients provided written informed consent before enrollment. The study was registered with ClinicalTrials.gov (identifier: NCT04989556 https://clinicaltrials.gov/study/NCT04989556) on 8 April 2021. The trial protocol and statistical analysis plan have been previously reported. Patients and members of the public were not involved in the design, analysis, or reporting of this secondary analysis, which utilized previously collected data from a randomized controlled trial.

### 2.2. Randomization and Blinding

Randomization evolved during study enrollment to accommodate changes in the trial design. Initially, patients were randomized to SPC alone or SPC+RSM with two slightly varied schedules of follow-up based on treatment type. Following a protocol amendment, these two SPC+RSM groups were combined due to the increasing use of combination therapies and because treatment could be addressed through stratified analyses. Patients were then randomized on a 1:1 basis. Randomization proceeded using permuted blocks of sizes 2 and 4 with stratification by age (18–39, 40–64, or ≥65 years) and/or cancer treatment (immunotherapy or non-immunotherapy) [17]. Treatment allocation was confirmed by an unblinded study coordinator based on assignments provided by a secure institutional website. Outcome assessments were conducted by a separate, blinded study coordinator. For this secondary analysis of acute care utilization outcomes, reviewers were blinded to treatment assignment during chart review. 

### 2.3. Intervention

The interventions have been previously described [17]. Briefly, both the SPC alone group and the SPC+RSM group received standard SPC delivered by an interdisciplinary team with scheduled in-person or virtual visits approximately once every four weeks throughout the study period. The first visit occurred within four weeks of study enrollment. Patients could contact the palliative care team via telephone or electronic patient portal as needed between clinic visits. Patients randomized to the SPC+RSM group received a weekly ESAS assessment via their patient portal, followed by a scheduled telephone call from a palliative care research coordinator twenty-four hours later to review symptoms, interim events, and other concerns for a total of 12 weeks from study enrollment. High-alert ESAS scores (8 or higher in pain, depression, anxiety, or shortness of breath) triggered escalation to a research nurse who would review the findings with an advanced practice provider and relay the management plan back to the patient. Any other clinically relevant changes in the ESAS would still be addressed during the scheduled telephone call.

### 2.4. Data Collection

Baseline demographic data were collected at enrollment, including age, sex, race, and cancer site. As stated previously, protocolized RSM was delivered weekly for 12 weeks starting from randomization. For our secondary analysis, a palliative care physician blinded to treatment assignment reviewed clinical charts to determine the dates and number of ED visits and/or hospitalizations for 6 months starting from study randomization. We sought to capture both unplanned and planned hospitalizations. ED visits leading to hospitalizations were considered discrete events for the purposes of analysis. In this analysis, an “encounter” is an individual ED visit or hospitalization. The reviewer then determined the primary presenting concern, the etiology of the presenting concern, and overall survival. Diagnoses were grouped into broad categories of etiology: disease progression, acute complications, treatment-related complications, and worsening symptoms (without clear disease progression). A single most likely etiology was determined for each acute care encounter. The etiology was categorized as disease progression if the patient had evidence of worsening disease on associated imaging or other evaluation (e.g., pain due to new metastases). Acute complications were acute medical events occurring in the setting of advanced cancer that were not clearly attributable to disease progression or cancer-directed therapy, such as pneumonia, pulmonary embolism, or heart failure exacerbation. Treatment-related complications were those caused directly by cancer treatment. Worsening symptoms without clear disease progression were those caused by a patient’s existing cancer burden rather than worsening disease (e.g., uncontrolled pain without change in tumor burden). 

The reviewer then classified an ED visit or hospitalization as potentially avoidable if the event could have been prevented by a palliative care RN telephone call followed by appropriate outpatient management [18]. Due to the inherent subjectivity, potential avoidability was then independently reviewed by a second palliative care physician. Determinations between the evaluators were then compared to assess interrater agreement. Discordant cases were reviewed by a third physician with both palliative and oncologic training who then made the final determination. Overall, 80/84 (95%) cases had congruent potentially avoidable determinations for ED visits and 55/57 (96%) for hospitalizations. Intervention-related adverse events were not separately assessed in this secondary analysis.

### 2.5. Statistical Analysis

All patients were analyzed on an intention-to-treat basis from randomization. All 100 patients randomized in the parent trial were included in the secondary analysis. Therefore, no imputation or other methods for handling missing data were performed. Sample size justification has been reported previously for the primary trial [17]. This post hoc secondary analysis used the 100 patients enrolled in the parent randomized trial, which was originally powered for symptom outcomes rather than acute care utilization [17]. No formal sample size calculation was performed for these outcomes, and the study may have been underpowered to detect modest between-group differences. Baseline characteristics were summarized using means and standard deviations for continuous variables and frequencies and percentages for categorical variables. Median overall survival (in days) and its 95% confidence interval were also reported. Between-group comparisons of baseline characteristics were descriptive, with assessment of balance based on clinical relevance. The primary outcomes were the relative frequencies of ED visits and hospitalizations between the SPC with RSM and SPC alone groups. Emergency department (ED) visits and hospitalizations were each evaluated as an ordinal categorical outcome (0, 1, 2, ≥3 visits) and compared between groups using Fisher’s exact test. Utilization was also summarized by group using the mean, standard deviation, median, and interquartile range. Group differences in the mean number of ED visits, avoidable ED visits, and hospitalizations were evaluated using two-sample *t*-tests with mean differences and 95% confidence intervals reported. Monthly ED visit and hospitalization rates were low and highly skewed; therefore, groups were compared using the Wilcoxon rank-sum test. We also performed a Wilcoxon rank-sum test when comparing the frequency of acute care utilization when treating an ED visit leading to hospitalization as a single event. We also examined the frequency and percentages of the main presenting concern, presenting concern etiology, and potential avoidability for each ED visit and hospitalization. All tests were two-sided, and a significance level of 0.05 was used for statistical inference. All analyses were conducted using Stata/SE version 18 (StataCorp LLC, College Station, TX, USA).

## 3. Results

### 3.1. Patient Characteristics

Among 100 enrolled patients, 43 (43%) were randomized to SPC alone, and 57 (57%) received SPC+RSM. The mean age was 56 (standard deviation [SD] 12), 63 patients (63%) were female, and 84 patients (84%) were White. The mean ESAS GDS was 33 (SD 13) and median overall survival was 221 days (95% CI, 171–271). See Table 1 for baseline patient characteristics.

### 3.2. Acute Care Utilization

During the 6-month observation period, patients experienced a total of 84 ED visits and 57 hospitalizations. Among patients assigned to SPC+RSM, there were 52 ED visits and 34 hospitalizations, corresponding to mean rates of 0.9 and 0.6 encounters per patient, respectively. Among patients assigned to SPC alone, there were 32 ED visits and 23 hospitalizations, corresponding to mean rates of 0.7 and 0.5 encounters per patient, respectively.

Between the SPC alone and SPC+RSM groups, no significant difference was observed in average ED visits per month (0.26 vs. 0.36, z = −0.82, *p* = 0.41). Similarly, average hospitalizations per month did not differ significantly between groups (0.20 vs. 0.26, z = 0.09, *p* = 0.93). 

Table 2 shows acute care utilization by treatment group. Among the 57 patients in the SPC+RSM group, there were 52 ED visits and 51 (98%) of these were unavoidable over the 6-month observation period. Among the 43 patients in the SPC alone group, there were 32 ED visits and 29 (91%) of these were unavoidable. Table 2 shows that there was no significant difference between SPC+RSM and SPC alone in the number of total ED visits (*p* = 0.58) and avoidable ED visits (*p* = 0.72).

**Figure 2:**
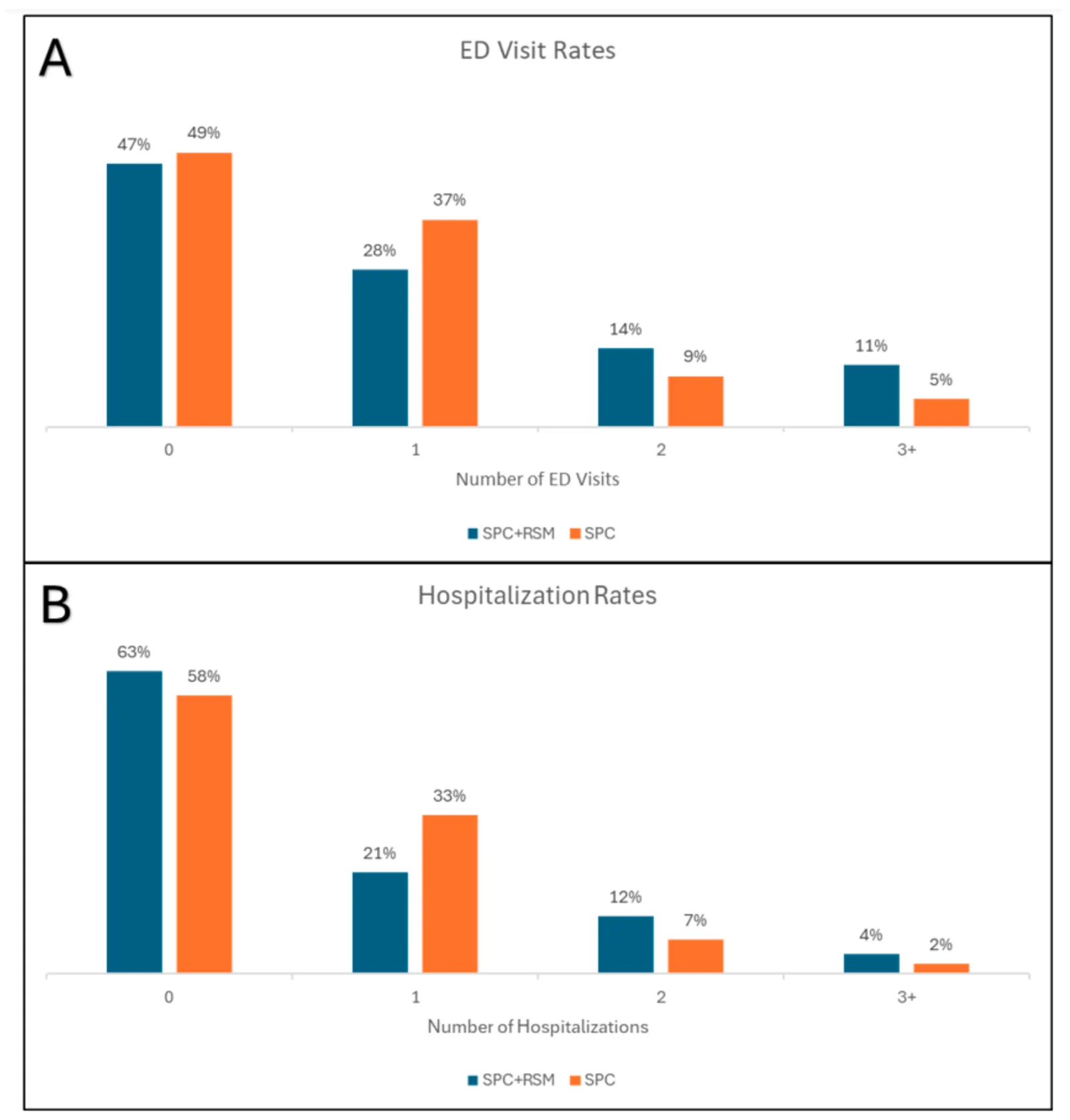
Distribution of emergency department (ED) visits (**A**) and hospitalizations (**B**) over 6 months among patients receiving specialist palliative care plus remote symptom monitoring (SPC+RSM; *n* = 57) versus specialist palliative care alone (SPC; *n* = 43). Bars represent the percentage of patients in each group with 0, 1, 2, or ≥3 visits. There were no significant differences between groups in the distribution of ED visits (*p* = 0.58) or hospitalizations (*p* = 0.56).

In the SPC+RSM group, there were 34 hospitalizations and none were avoidable. In the SPC group, there were 23 hospitalizations and none were avoidable. There were 34 hospitalizations in the SPC+RSM group and 23 hospitalizations in the SPC alone group. Table 2 shows that there was no significant difference between SPC+RSM and SPC alone in the number of total hospitalizations (*p* = 0.56). No avoidable hospitalizations occurred in either group. Figure 2 shows the distribution of ED visits and hospitalizations in the SPC+RSM versus SPC alone groups. All hospitalizations were unplanned, with no planned hospitalizations observed during the 6-month observation period. 

We also performed a sensitivity analysis which treated an ED visit leading to hospitalization as a single encounter. We found no significant difference in the average number of encounters between the SPC+RSM and SPC alone groups (Mean = 0.91 vs. 0.79, z = −0.38, *p* = 0.71). 

### 3.3. Reasons for Acute Care Utilization

The most common presenting concerns leading to ED visits were pain (27%), respiratory symptoms (22%), and abnormal laboratory or imaging findings (15%) (Table 3). Hospitalizations were similarly driven by pain (29%), respiratory symptoms (22%), and constitutional symptoms (16%).

**Figure 3:**
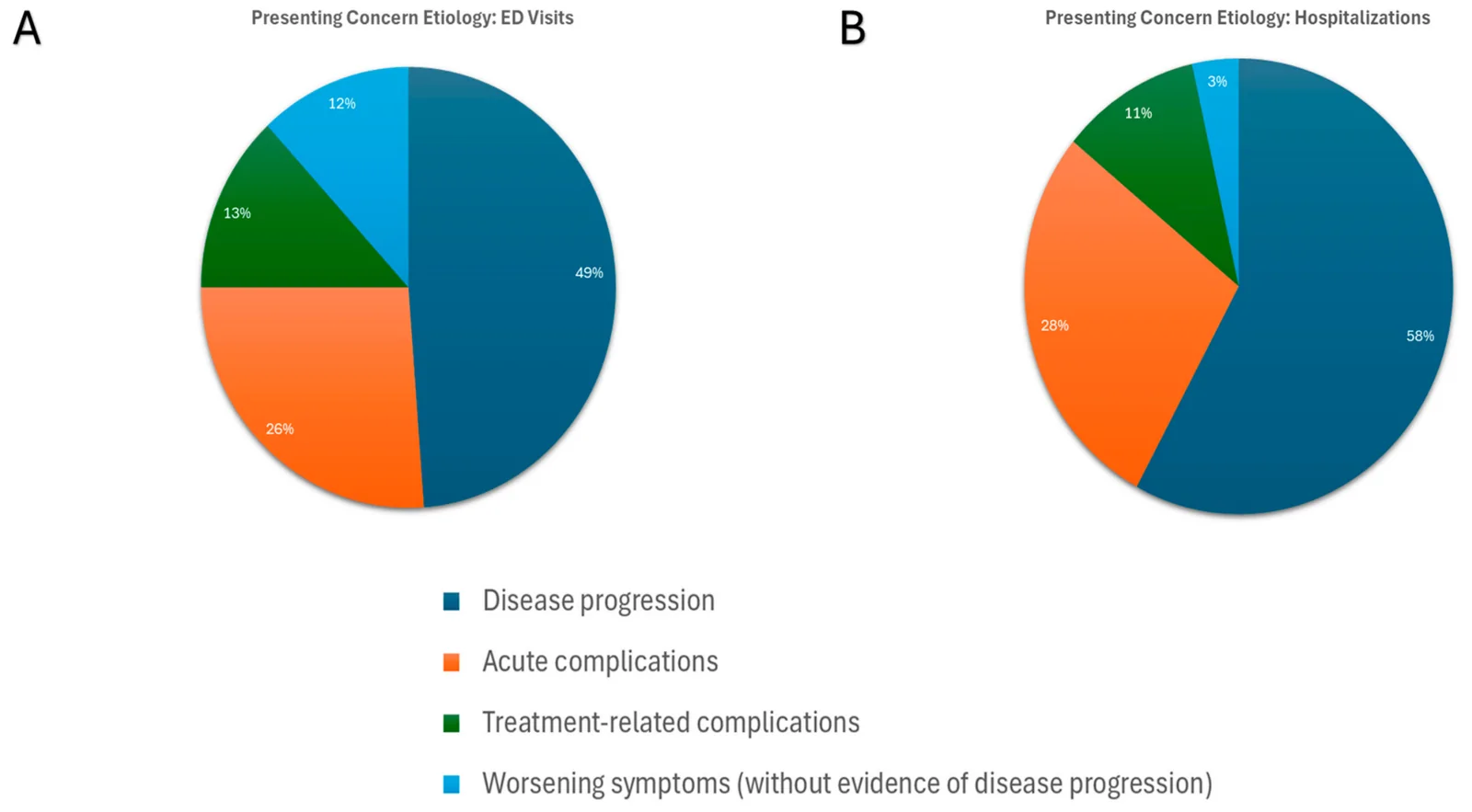
Etiologies of emergency department visits (**A**) and hospitalizations (**B**) over 6 months among patients with advanced cancer enrolled in phase I clinical trials. Disease progression was the most common etiology followed by acute complications for both ED visits and hospitalizations.

When we examined the etiology of the main presenting concern, disease progression accounted for approximately half of ED visits (49%) and most hospitalizations (58%). This was followed by acute complications (ED 26%, hospitalizations 28%), treatment-related complications (ED 13%, hospitalizations 11%), and worsening symptoms without clear disease progression (ED 12%, hospitalizations 3%) (Figure 3).

## 4. Discussion

In this secondary analysis of a randomized clinical trial evaluating RSM integrated into SPC for patients with advanced cancer undergoing phase I therapies, we found high rates of ED visits and hospitalizations over the 6-month observation period. Despite evidence from the parent trial suggesting an improvement in health-related quality of life with RSM integration, this did not result in statistically significant differences in acute care utilization or overall survival compared with SPC alone. Notably, few ED visits and no hospitalizations were deemed potentially avoidable under our retrospective assessment criteria. Disease progression was the most common etiology of both ED visits and hospitalizations. These findings suggest that although RSM may improve patient-reported outcomes, acute care utilization in this population may be driven primarily by disease biology and clinical trajectory.

Few studies have examined acute care utilization in cancer patients undergoing phase I therapies. In our cohort, 52% of patients presented to the ED and 39% required hospitalization over 6 months, reflecting a substantial burden of acute care utilization. Previous studies have reported unplanned hospitalizations in 22.6% of phase I patients during selected 2-month observation windows and 31.7% over 6 months in patients participating in a randomized trial of a palliative care intervention [6,7]. Although differences in study design, follow-up duration, and patient populations limit direct comparisons, these findings are consistent with the significant burden of acute care utilization observed in our cohort. Several factors may have influenced our observed rates. Patients were fit enough to participate in both a phase I oncology and RSM clinical trial simultaneously, potentially representing a fitter subset of the broader phase I population. Similarly, involvement of the palliative care team has repeatedly been shown to reduce acute care utilization in cancer patients [10,11,19]. Conversely, our inclusion criteria required moderate-to-high symptom burden, possibly selecting for patients at greater risk of acute care utilization.

Although there are limited data on acute care utilization in phase I patients, rates from patients undergoing non-phase I clinical trials appear to be lower. For example, one 2024 cohort study of cancer patients aged 65 years or older participating in an oncologic clinical trial found that 36.1% had an ED visit and 32.4% were hospitalized within 12 months of trial enrollment—lower rates of acute care utilization despite a longer follow-up period compared to our cohort [20]. Additionally, our data are consistent with studies examining acute care utilization at the end of life, including a systematic review which found ED utilization rates between 48% and 91% in the last 6 months [21]. This similarity is not unexpected given the limited prognosis of our cohort, which had a median survival of approximately seven months. Taken together, these data suggest a relatively high burden of acute care utilization in our cohort.

We did not detect a statistically significant difference in these high rates of acute care utilization with the addition of RSM in this clinical trial. Findings in non-phase I settings across the literature have been mixed, with some studies showing benefits while others show no benefit or even worse outcomes [12,13,14,15,22,23,24,25]. Furthermore, it is difficult to draw definitive conclusions due to heterogeneity in study design and implementation. One explanation for the lack of benefit observed in our cohort may be the intensive clinical surveillance which patients already received through both the investigational oncology and palliative care teams, limiting the incremental benefit of further monitoring. Another reason could be insufficient monitoring frequency—perhaps RSM needs to be administered more than weekly to be effective. More fundamentally, RSM is designed to identify patients in distress and trigger timely clinical action, but it cannot itself alter the underlying disease trajectory or prevent all acute complications. Although earlier detection may facilitate outpatient management for some symptoms, patients with progressive disease or acute complications may still require presentation to the ED and/or hospitalization. The predominance of these etiologies may therefore have limited the capacity of RSM to reduce acute care utilization in this population. 

Indeed, a more granular review of our data revealed that few ED visits and no hospitalizations were deemed potentially avoidable using a timely RSM-initiated intervention, consistent with the high acuity of phase I cancer patients. In fact, the proportion of acute care encounters classified as potentially avoidable in the general cancer population ranges from 10% to52%, much higher than those found in our study, although differences in patient populations and assessment criteria limit direct comparisons [18,26,27,28,29,30,31]. Disease progression was the most common cause of both ED visits and hospitalizations in our analysis, followed by acute complications. Although these findings highlight the substantial burden of advanced disease in this population, the underlying etiology of an ED visit or hospitalization does not necessarily determine its preventability.

These findings have important implications for the care of patients undergoing phase I therapies. The elevated rates of acute care utilization observed despite integrated SPC suggest that clinicians should anticipate frequent clinical deterioration in this population and incorporate proactive discussions regarding prognosis, goals-of-care, and advance care planning early in the disease trajectory. Rather than focusing solely on preventing ED visits or hospitalizations, care models may need to emphasize preparation for these events and alignment of treatment decisions with patient priorities. These results also suggest that interventions aimed solely at increasing symptom monitoring may be insufficient in this population, and that alternative strategies—such as anticipatory care planning, hospital-at-home models, or dedicated oncology urgent care pathways—may be necessary to better support this high-risk group [32,33,34]. More research is needed to determine if these interventions can support phase I patients.

This study has several limitations that warrant consideration. This was a post hoc secondary analysis of a randomized trial that was originally powered to evaluate symptom outcomes rather than acute care utilization. Therefore, any findings should be interpreted as hypothesis-generating. The small sample size may have limited our ability to detect modest differences between groups. Because the parent trial was designed to evaluate symptom outcomes rather than acute care utilization, no formal sample size calculation was performed for ED visits or hospitalizations. However, the absolute between-group difference in acute care utilization was small (approximately 5 percentage points or less) and likely of limited clinical significance in our study. Although potential avoidability assessments were performed by independent reviewers and interrater agreement was evaluated with a third reviewer arbitrating the final determination, the classification of encounters as potentially avoidable remains inherently subjective and may be influenced by retrospective interpretation. Furthermore, data regarding assessment completion, telephone-call adherence, alerts and subsequent clinical actions were not collected. We were also unable to examine encounters that occurred outside of MD Anderson. If patients sought care elsewhere, our data would represent an underestimate of the already high rates of observed acute care utilization. Additionally, standardized symptom assessments like ESAS are unable to capture the complete spectrum of clinically significant symptoms experienced by patients. However, we sought to mitigate this by ensuring weekly follow-up regardless of symptom index severity. Finally, this study was conducted at a single tertiary cancer center with a highly selected cohort of patients who were eligible for phase I therapy, had moderate-to-high symptom burden, and received SPC with intensive trial monitoring. Consequently, rates and drivers of acute care utilization may differ in broader advanced cancer populations, including patients receiving standard therapies, those treated in community settings, and patients not receiving SPC or the increased monitoring associated with clinical trial enrollment. 

## 5. Conclusions

In conclusion, phase I patients had high rates of ED visits and hospitalizations even when they received SPC in this post hoc secondary analysis. We did not observe a statistically significant difference in acute care utilization with the addition of RSM in this cohort. Few ED visits and no hospitalizations were classified as potentially avoidable according to our retrospective assessment criteria. Acute care encounters were most frequently attributed to disease progression and acute complications. While RSM improved symptom burden and health-related quality of life in the parent trial, these exploratory findings highlight the need for novel strategies to support phase I cancer patients as they navigate the acute care system.

## Figures and Tables

**Table 1:** Baseline patient characteristics.

	Total *n* = 100 (%) ^a^	SPC+RSM *n* = 57 (%) ^a^	SPC Alone *n* = 43 (%) ^a^
Age, mean (SD)	56 (12)	56 (13)	56 (12)
Sex			
Female	63 (63)	37 (65)	26 (60)
Male	37 (37)	20 (35)	17 (40)
Race			
White or Caucasian	84 (84)	48 (84)	36 (84)
Black or African American	9 (9)	5 (9)	4 (9)
Asian	5 (5)	4 (7)	1 (2)
American Indian or Alaska Native	2 (2)	0 (0)	2 (5)
Ethnicity			
Non-Hispanic/non-LatinX	89 (89)	50 (88)	39 (91)
Hispanic/LatinX	11 (11)	7 (12)	4 (9)
Cancer site			
Gastrointestinal	33 (33)	15 (26)	18 (42)
Gynecologic	19 (19)	13 (23)	6 (14)
Other	16 (16)	10 (18)	6 (14)
Breast	14 (14)	9 (16)	5 (11)
Genitourinary	9 (9)	6 (10)	3 (7)
Respiratory	6 (6)	4 (7)	2 (5)
Head and neck	3 (3)	0 (0)	3 (7)
Immunotherapy allocation			
Immunotherapy	19 (19)	8 (14)	11 (26)
Non-Immunotherapy	81 (81)	49 (86)	32 (74)
Edmonton Symptom Assessment System ^b^			
Global Distress Score, mean (SD) ^c^	33 (13)	31 (13)	35 (13)
Pain, mean (SD)	5 (3)	5 (3)	5 (3)
Fatigue, mean (SD)	6 (2)	5 (2)	6 (2)
Nausea, mean (SD)	2 (3)	2 (3)	2 (2)
Drowsiness, mean (SD)	3 (3)	3 (3)	4 (3)
Appetite, mean (SD)	4 (3)	4 (3)	5 (3)
Dyspnea, mean (SD)	3 (3)	2 (3)	3 (3)
Depression, mean (SD)	2 (2)	2 (2)	2 (2)
Anxiety, mean (SD)	3 (3)	3 (3)	3 (3)
Well-being, mean (SD)	5 (2)	4 (2)	5 (3)
Sleep, mean (SD)	5 (2)	5 (2)	5 (3)
Overall survival in days, median (95% CI)	221 (171, 271)	222 (152, 425)	208 (141, 264)

Abbreviations: CI, confidence interval; RSM, remote symptom monitoring; SD, standard deviation; SPC, specialist palliative care. ^a^ Values are *n* (%) unless otherwise indicated. ^b^ The Edmonton Symptom Assessment System is a validated, self-administered tool that assesses patient symptoms using a visual analogue scale that ranges from 0–10 for each symptom, where 0 = no symptoms and 10 = worst symptom burden. ^c^ The Global Distress Score is a subscale of the first nine physical and psychosocial Edmonton Symptom Assessment System symptoms and ranges from 0–90, with lower scores indicating better symptom control.

**Table 2:** Emergency Department Visits and Hospitalizations by Treatment Group.

	SPC+RSM *n* = 57 (%) ^a^	SPC Alone *n* = 43 (%) ^a^	Mean Difference 95% CI	*p*-Value ^b^
Total Number of ED Visits				
0	27 (47)	21 (49)		0.58
1	16 (28)	16 (37)		
2	8 (14)	4 (9)		
3+	6 (11)	2 (5)		
Mean (SD)	0.9 (1.1)	0.7 (1.0)	0.2 (−0.3, 0.6)	
Median (Q1, Q3)	1 (0.0–1.0)	1 (0.0–1.0)		
Number of Avoidable ED Visits				
0	56 (98)	41 (95)		0.72
1	1 (2)	1 (2)		
2	0 (0)	1 (2)		
3+	0 (0)	0 (0)		
Mean (SD)	0.0 (0.1)	0.1 (0.3)	−0.1 (−0.1, 0)	
Median (Q1, Q3)	0.0 (0.0–0.0)	0.0 (0.0–0.0)		
Total Number of Hospitalizations				
0	36 (63)	25 (58)		0.56
1	12 (21)	14 (33)		
2	7 (12)	3 (7)		
3+	2 (4)	1 (2)		
Mean (SD)	0.6 (1.0)	0.5 (0.7)	0.1 (−0.3, 0.4)	
Median (Q1, Q3)	0.0 (0.0–1.0)	0.0 (0.0–1.0)		
Number of Avoidable Hospitalizations				
0	57 (100)	43 (100)		**-**
1	0 (0)	0 (0)		
2	0 (0)	0 (0)		
3+	0 (0)	0 (0)		
Mean (SD)	0 (0)	0 (0)	0 (0, 0)	
Median (Q1, Q3)	0.0 (0.0–0.0)	0.0 (0.0–0.0)		

Abbreviations: ED, emergency department; RSM, remote symptom monitoring; SD, standard deviation; SPC, specialist palliative care. ^a^ Values are *n* (%) unless otherwise indicated. ^b^ Fisher’s exact test was used to compare categorical distributions between treatment groups.

**Table 3:** Reasons for Emergency Department Visits and Hospitalizations by Encounter Type.

	Emergency Department Visits *n* = 84 (%) ^a^	Hospitalizations *n* = 57 (%) ^a^
Main presenting concern		
Pain	23 (27)	17 (29)
Respiratory symptoms	19 (22)	13 (22)
Abnormal labs/imaging	13 (15)	7 (12)
Constitutional symptoms	10 (12)	9 (16)
Nausea	5 (6)	2 (4)
Delirium	3 (4)	3 (5)
Bleed	3 (4)	2 (4)
Neurological symptoms	3 (4)	1 (2)
Other	3 (4)	1 (2)
Diarrhea	2 (2)	2 (4)
Presenting concern etiology		
Disease progression	41 (49)	33 (58)
Acute complications	22 (26)	16 (28)
Treatment-related complications	11 (13)	6 (11)
Worsening symptoms (without evidence of disease progression)	10 (12)	2 (3)
Avoidable encounter		
No	80 (95)	57 (100)
Yes	4 (5)	0 (0)

^a^ Total number of acute care encounters during the 6-month observation period for the combined SPC+RSM and SPC alone cohort. Some patients had more than one encounter.

## Data Availability

Investigators’ proposed use of the data must be approved by the Institutional Review Board at the University of Texas MD Anderson Cancer Center. Proposals should be directed to dhui@mdanderson.org. To gain access, data requestors will need to sign a data access agreement.

## Abbreviations

The following abbreviations are used in this manuscript:

ED	Emergency department
RSM	Remote symptom monitoring
SPC	Specialist palliative care
ESAS	Edmonton Symptom Assessment System
GDS	Global Distress Score
MDAS	Memorial Delirium Assessment Scale
